# Clinical risk factors and genomic characterization of carbapenem-resistant *Klebsiella pneumoniae* infections in a traditional Chinese medicine hospital

**DOI:** 10.3389/fcimb.2026.1738197

**Published:** 2026-05-25

**Authors:** Wenxia Zhang, Shufen Liu, Boyu Yang, Xiuhua Hu, Minlan Zhang, Xinghua Zhou, Jinrong Guo, Min He, Fenghua Zhang

**Affiliations:** 1 Department of Clinical Laboratory, Shanghai University of Medicine and Health Science Affiliated Zhoupu Hospital, Shanghai, China; 2 Clinical Research Center, Shuguang Hospital, Shanghai University of Traditional Chinese Medicine, Shanghai, China; 3 Department of Traditional Chinese Medicine, Shanghai University of Medicine & Health Sciences Affiliated Zhoupu Hospital, Shanghai, China

**Keywords:** *bla*_KPC-2_ variant, *Klebsiella pneumoniae*, mortality risk factors, virulence factors, whole genome sequencing

## Abstract

**Background:**

Carbapenem-resistant *Klebsiella pneumoniae* (CRKP), particularly hypervirulent multidrug-resistant strains, has become a major cause of healthcare-associated infections with high mortality. However, molecular characteristics and mortality risk factors for CRKP infections in Chinese Traditional Medicine hospitals remain poorly understood.

**Methods:**

We conducted a retrospective study of patients with microbiologically confirmed CRKP infections at a tertiary TCM hospital between 2019 and 2022. Multivariate logistic regression was used to assess independent risk factors for mortality. We performed whole-genome sequencing on 102 CRKP isolates (2022) to characterize antimicrobial resistance determinants, virulence factors, and phylogenetic relationships.

**Results:**

In our cohort of 233 CRKP-infected patients (76 non-survivors, 157 survivors), multivariate analysis identified four independent predictors of in-hospital mortality: multiple organ dysfunction syndrome (aOR 3.69, 95% CI 1.28-10.61; P = 0.015), sepsis (aOR 3.04, 95% CI 1.30-7.12; P = 0.011), invasive mechanical ventilation (aOR 5.95, 95% CI 2.78-12.75; P<0.001), and ICU admission (aOR 4.10, 95% CI 1.55-10.84; P = 0.005). Genomic analysis identified ST11-KL64 (55.9%, 57/102) and ST15-KL19 (21.6%, 22/102) as dominant clones, alongside novel strains ST1658-KL60 and ST3345-KL8. The prevalent plasmid was IncFIB(K) (85.3%), followed by IncHI1B (66.7%); IncFII(pHN7A8) (28.4%) was exclusive to ST11. The predominant capsular type was KL64:O2a. Carbapenem resistance was primarily driven by *bla*_KPC-2_ (92.2%, 94/102). In addition to the dominant wild-type *bla*_KPC-2_, we identified two other *bla*_KPC-2_ variants: one strain harboring *bla*_KPC-33_, and one strain harboring a novel *bla*_KPC-2_ variant with an L169T substitution (CTG→ACG) in the Ω-loop domain, which confers ceftazidime-avibactam resistance. Eighty-two isolates (80.4%) exhibited high virulence (score 4), characterized by aerobactin/salmochelin co-occurrence with yersiniabactin (*ybt* 9/ICE*Kp3* predominant), but lacking colibactin.

**Conclusions:**

Our study highlights the predominance of ST11-KL64 and ST15-KL19 CRKP clones in a TCM hospital setting, with *bla*_KPC-2_ as the main driver of carbapenem resistance. High virulence and multidrug resistance are common features, contributing to significant mortality. These findings underscore the need for targeted infection control and treatment strategies in TCM hospitals.

## Introductions

*Klebsiella pneumoniae* is a leading cause of severe infections, including pneumonia, sepsis, urinary tract infections, and soft tissue infections (Zou and Li, 2021; Dong et al., 2024; Wang et al., 2024; Yu et al., 2024). Notably, *K. pneumoniae* has evolved into two distinct pathotypes: classical *K. pneumoniae* (cKp) and hypervirulent *K. pneumoniae* (hvKp). cKp is typically associated with hospital-acquired infections in immunocompromised or debilitated hosts and is known for its ability to acquire multiple antimicrobial resistance genes. By contrast, hvKp was initially associated with community-acquired infections in healthy individuals, with a particular propensity for causing metastatic infections such as endophthalmitis and pyogenic liver abscess (Tang et al., 2025).

In recent years, carbapenem-resistant *Klebsiella pneumoniae* (CRKP) has spread globally, emerging as a significant worldwide public health threat. According to the most recent official data from the China Antimicrobial Surveillance Network (CHINET), the rate of imipenem-resistant *K. pneumoniae* in China has risen steadily from 2.9% in 2005 to 22.6% in 2024(https://www.chinets.com/Data/GermYear), severely limiting therapeutic options. Reflecting this threat, the World Health Organization (WHO) has still classified CRKP as a critical-priority antimicrobial-resistant pathogen in its 2024 report (https://www.who.int/publications/i/item/9789240093461). Moreover, *K. pneumoniae* serves as a crucial reservoir for resistance genes, which are frequently disseminated via plasmid-mediated horizontal gene transfer, accelerating the spread of antibiotic resistance in clinical settings (Göpel et al., 2025).

Of particular concern is the emergence of hypervirulent CRKP (hv-CRKP) strains, such as ST11, which acquire virulence plasmids, enhancing their pathogenicity (Gu et al., 2018). A longitudinal multi-centre study in China revealed that the prevalence of hv-CRKP increased significantly from 28.2% in 2016 to 45.7% in 2020 (Hu et al., 2024). These hv-CRKP strains exhibit multi-drug resistance, enhanced virulence, and extremely limited therapeutic options, resulting in significantly higher mortality rates (Kohler et al., 2017; Li et al., 2023).

However, the molecular epidemiology and mortality risk factors associated with CRKP infections vary substantially across regions and healthcare settings. In China, CRKP infections are predominantly caused by ST11-KL64 strains carrying *bla*_KPC-2_, *bla*_CTX-M-65_, and virulence genes like *iucA* and *rmpA2*, which are rarely detected in other countries (Wang et al., 2022). Furthermore, KL64 was the predominant capsule serotype in eastern and central China (e.g., Shanghai, Zhejiang, Sichuan), while KL47 was more common in northern and northeastern regions (e.g., Beijing, Liaoning, Tianjin) (Hu et al., 2024). Resistance mechanisms also differ by patient demographics. In China, *bla*_NDM_ is more common in pediatric populations, whereas *bla*_KPC_ predominates in adults (Han et al., 2020; Ran et al., 2025). Despite these insights, the data on CRKP molecular characterization and mortality risk factors in TCM hospitals remain scarce in China.

Here, we analyzed a cohort of CRKP-infected patients from a tertiary TCM hospital in Shanghai (2019–2022) to identify risk factors for in-hospital mortality. Furthermore, we performed whole-genome sequencing (WGS) on 2022 CRKP isolates to elucidate their molecular epidemiology and resistance mechanisms. Our findings aim to guide clinical management and inform infection control strategies against CRKP in TCM hospitals.

## Methods

### Study design and patient population

This single-center retrospective observational study was conducted at a tertiary hospital, a 1200-bed Traditional Chinese Medicine (TCM) medical center in Shanghai, China. We included all hospitalized patients with microbiologically confirmed CRKP infections between January 2019 and December 2022. CRKP infection was defined according to the general principles outlined in the Chinese national standard for diagnosis of healthcare-associated infections (WS/T 857-2025). Specifically, infection was determined by a comprehensive review of medical records by two independent investigators, requiring: (1) isolation of CRKP from a normally sterile site, or from a non-sterile site accompanied by compatible clinical signs and symptoms of infection (e.g., fever, leukocytosis, local inflammatory signs), and (2) the initiation of targeted antibiotic therapy by the treating physician. CRKP colonization was defined as the isolation of CRKP in the absence of compatible clinical symptoms or signs, or when the clinical presentation was clearly attributable to another etiology, and no anti-CRKP treatment was initiated.

Accordingly, we excluded patients with: (1) incomplete medical records, (2) CRKP colonization (as defined above) without infection, or (3) polymicrobial infections where CRKP was not the primary pathogen.

Clinical and microbiological data were extracted from the medical records. Epidemiological and clinical data, including demographics, comorbidities, invasive procedures, outcomes, and sources of culture, were collected. Patients were then categorized into survival and non-survival groups based on their outcomes to analyze the risk factors associated with in-hospital mortality in patients with CRKP infections.

In 2022, we performed active surveillance through rectal swab screening of hospitalized patients. From these screenings and clinical cultures, we obtained 102 non-duplicate CRKP isolates for whole-genome sequencing analysis. All isolates were preserved at -80 °C and subcultured for further study. The study protocol was approved by the Ethics Committee of Shuguang Hospital, Shanghai University of Traditional Chinese Medicine (Approval No. 2023-1332-99-01). As this work represents a collaborative effort between Shuguang Hospital and Zhoupu Hospital (Shanghai University of Medicine and Health Sciences), the protocol was also acknowledged by the Ethics Committee of Shanghai Zhoupu Hospital (Approval No. 2024-C-012-E01) for administrative purposes related to the current affiliation of the first author.

### Bacterial identification and MIC determination

The identification and antimicrobial susceptibility testing of the strains were conducted using the VITEK-2 automated microbial analysis system (bioMérieux, France). The minimum inhibitory concentrations (MICs) for ceftazidime/avibactam (CZA) and polymyxin B were determined by the microbroth dilution method, while the MIC for tigecycline was assessed using the E-test. The breakpoints for drug sensitivity and carbapenem resistance were interpreted in accordance with the CLSI 2021 guidelines from the American Clinical Laboratory Standardization Institute. The tigecycline breakpoint was interpreted according to the European Committee on Antimicrobial Susceptibility Testing (EUCAST) standards, available at EUCAST Clinical Breakpoints. Escherichia coli ATCC 25922, provided by the Shanghai Centre for Clinical Laboratory, served as the quality control strain.

### Whole-genome sequencing and genomic characterization

WGS was performed on 102 CRKP isolates collected in 2022. Sequencing was performed on an Illumina NovaSeq 6000 platform with an average of 10 million reads per isolate using 150-base paired-end reads. All sequences were uploaded to the NCBI with Bioproject (PRJNA1233633). Core SNPs were used to create a phylogenetic tree using Pathogenwatch (https://pathogen.watch/). Sequence types (ST), were performed using *Klebsiella pneumoniae*-Pasteur, and surface polysaccharide typing of polysaccharide capsule loci(KL) and lipopolysaccharide loci (O), were performed using Kaptive. Antimicrobial resistance(AMR) and virulence factors were analyzed using Kleborate. Plasmid replicon analysis was identified using the Plasmid Finder tool on the CGE server (https://cge.food.dtu.dk/services/MobileElementFinder/).

### Statistical analysis

Categorical variables were presented as frequencies and percentages, and continuous variables were expressed as mean ± standard deviation. Univariate analysis was performed using the chi-square test or Fisher’s exact test for categorical variables and Student’s t-test or Mann-Whitney U test for continuous variables, depending on data distribution. Variables with P < 0.05 in univariate analysis were included in a multivariable logistic regression model to identify independent risk factors for in-hospital mortality. Adjusted odds ratios (aORs) with 95% confidence intervals (CIs) were calculated. A two-tailed P<0.05 was considered statistically significant. All analyses were conducted using IBM SPSS Statistics (version 24.0).

## Results

### Bacterial characteristics

From 2019 to 2022, a total of 233 CRKP-infected patients were included in the study. The infections were predominantly identified in intensive care unit (ICU) patients (62.7%, 146/233), with lower frequencies observed in internal medicine (24.9%, 58/233) and surgical (12.4%, 29/233) departments.

**Figure 1:**
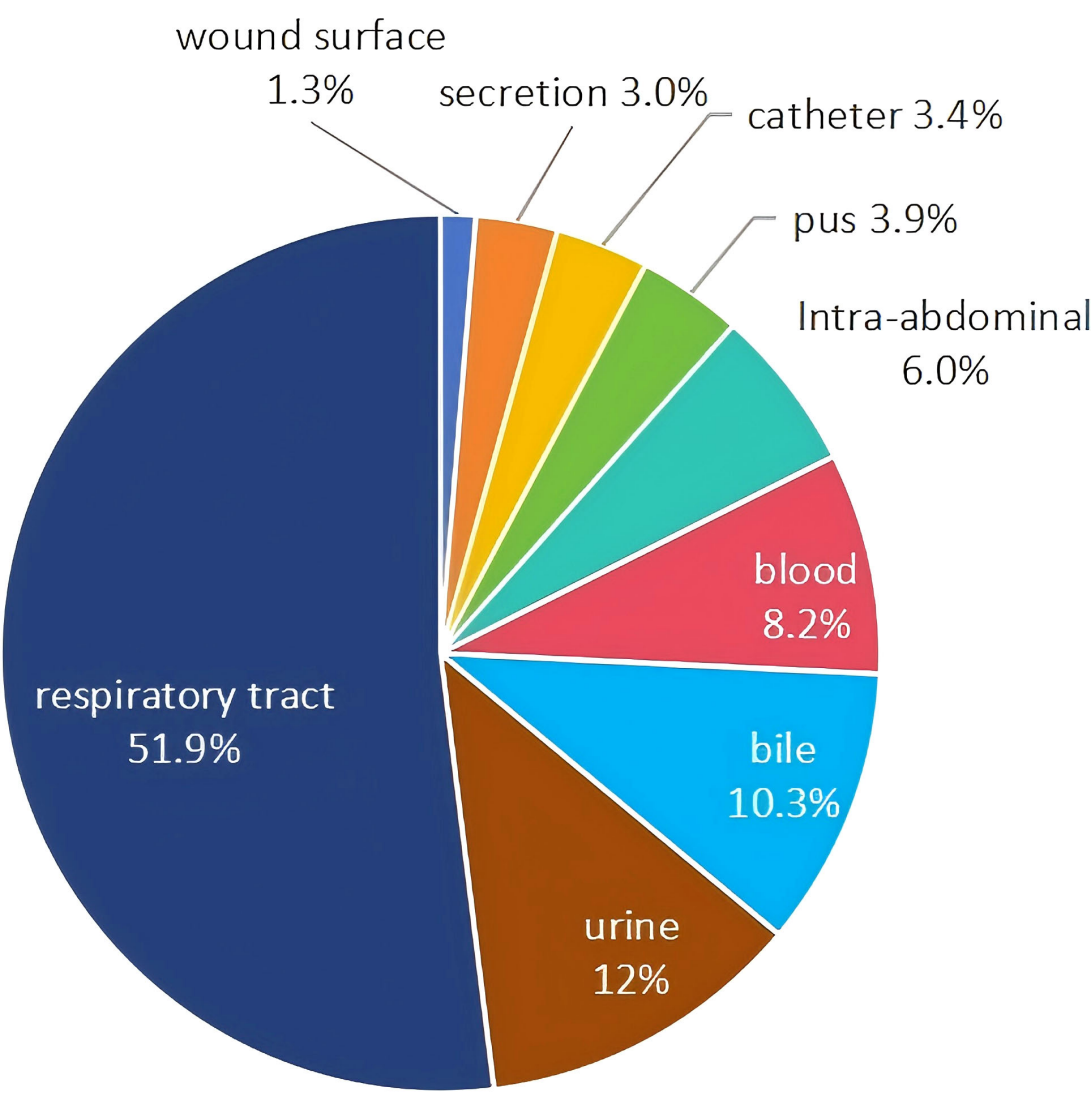
Distribution of clinical specimen sources for CRKP isolates (2019–2022).

As shown in **Figure 1**, respiratory tract specimens constituted the most common source of CRKP isolates (51.9%, 121/233), followed by urine (12.0%, 28/233), bile (10.3%, 24/233), and blood cultures (8.2%, 19/233).

### Antimicrobial resistance patterns

The carbapenem resistance rates among *K. pneumoniae* isolates showed significant temporal variations during the study period (2019-2022) **Supplementary Table 1**. The resistance rate decreased from 18.5% in 2019 to 14.5% in 2020, reaching its lowest point at 13.6% in 2021, before sharply increasing to 25.0% in 2022.

Despite this variability, CRKP isolates demonstrated consistently high resistance rates (>80%) to the majority of tested antimicrobial agents. The most notable exceptions were CZA, polymyxin B, and tigecycline, which maintained resistance rates below 10% throughout the study period. However, a progressive increase in resistance to CZA was observed, with resistance rates rising from 3.1% in 2020 to 9.6% in 2022.

### Risk factors for in-hospital mortality

During the 2019–2022 study period, 233 CRKP-infected patients were analyzed, comprising 76 non-survivors (32.6%) and 157 survivors (67.4%). The demographic and clinical characteristics of the cohort are summarized in **Table 1**. Univariate analysis identified several factors significantly associated with increased in-hospital mortality (P<0.05), including advanced age (OR 1.041, 95% CI 1.019–1.064), ICU admission (OR 7.350, 95% CI 3.427-15.763), multiple organ dysfunction syndrome (OR 6.208, 95% CI 2.573–14.977), pneumonia (OR 2.412, 95% CI 1.378-4.221), cardiovascular diseases (OR 2.359, 95% CI 1.299-4.280), sepsis (OR 5.628, 95% CI 2.733-11.591), gastric catheterization (OR 2.047, 95% CI 1.103-3.801), and invasive mechanical ventilation (OR 5.746, 95% CI 3.037-10.871).

**Table 1:** Univariate and multivariate analysis of risk factors for in-hospital mortality accociated with CRKP ifnection.

Variables	Survivors (n=157)	Non-survivals (n=76)	Univariate analysis		Multivariate analysis	
			OR (95% CI)	P value	OR (95% CI)	P value
Demographic						
Age (Years, mean±SD)	65.48 ± 16.37	73.63 ± 11.68	1.041 (1.019-1.064)	<0.001	1.051 (1.02-1.082)	0.001
days in hospital	25.10 ± 21.87	28.72 ± 25.69	1.006 (0.995-1.018)	0.268		
Gender Male, n (%)	111 (67.7)	53 (69.7)	0.955 (0.525-1.737)	0.880		
ICU Admission	79 (50.3)	67 (88.2)	7.350 (3.427-15.763)	<0.001	4.095 (1.548-10.835)	0.005
Underlying diseases or Comorbidity Index						
Diabetes mellitus	64 (40.8)	32 (42.1)	1.057 (0.606-1.842)	0.845		
Hypertension	95 (60.5)	49 (64.5)	1.184 (0.671-2.091)	0.559		
Multiple Organ Dysfunction Syndrome	8 (5.1)	19 (25)	6.208 (2.573-14.977)	<0.001	3.691 (1.284-10.608)	0.015
Pneumonia	57 (36.3)	44 (57.9)	2.412 (1.378-4.221)	0.002	1.281 (0.64-2.563)	0.485
Cardiovascular diseases	24 (16.3)	30 (39.5)	2.359 (1.299-4.28)	0.004	1.659 (0.801-3.436)	0.173
Hepatobiliary and pancreatic diseases	50 (31.8)	16 (21.1)	0.571 (0.299-1.088)	0.086		
Kidney diseases	28 (17.8)	21 (27.6)	1.759 (0.092-3.362)	0.085		
Cancer	17 (10.8)	10 (13.2)	1.248 (0.542-2.873)	0.602		
Sepsis	14 (8.9)	27 (35.5)	5.628 (2.733-11.591)	<0.001	3.04 (1.297-7.124)	0.011
Invasive procedures						
Electronic Bronchoscopy	10 (6.4)	10 (13.2)	2.227 (0.885-5.608)	0.083		
Central venous catheterization	14 (8.9)	9 (11.8)	1.372 (0.566-3.329)	0.483		
Gastric catheterization	96 (61.1)	58 (76.3)	2.047 (1.103-3.801)	0.022	0.48 (0.197-1.169)	0.106
Urinary catheterization	112 (71.3)	62 (81.6)	1.779 (0.906-3.496)	0.092		
Invasive Mechanical Ventilation	62 (39.5)	60 (78.9)	5.746 (3.037-10.871)	<0.001	5.948 (2.775-12.749)	<0.001

After adjusting for confounding factors in the multivariable model, multiple organ dysfunction syndrome (aOR 3.691, 95% CI 1.284-10.608, P = 0.015) and sepsis (aOR 3.04, 95% CI 1.297-7.124, P = 0.011) were independent predictors of in-hospital mortality compared to other underlying conditions. Similarly, invasive mechanical ventilation (aOR 5.948, 95% CI 2.775-12.749, P<0.001) and ICU admission (aOR 4.095, 95% CI 1.548-10.835, P = 0.005) were also significantly associated with an increased risk of death.

### Multilocus sequence typing

MLST analysis of 102 CRKP strains identified 8 distinct sequence types (STs). The dominant lineage was ST11 (65.7%, 67/102), followed by ST15 (21.6%, 22/102). Less prevalent types included ST29 (n=4), ST42 (n=3), and ST307 (n=3). Furthermore, ST1049, ST1658, and ST3345 were also identified, with the latter two being first reported (**Supplementary Table 2**).

Plasmids

**Figure 2:**
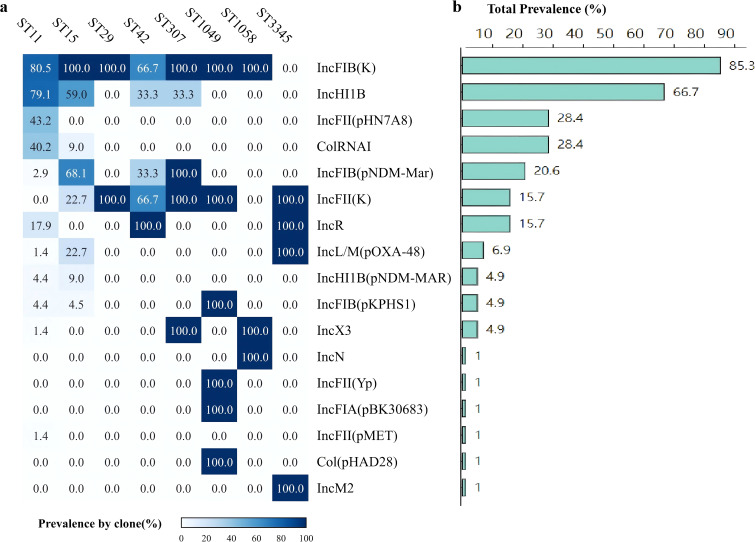
Plasmid diversity in 102 CRKP isolates. **(a)** The heatmap shows the prevalence of plasmid types within different bacterial clones, represented by ST (sequence type). The color gradient indicates the percentage of prevalence, with darker shades representing higher percentages. **(b)** The bar chart shows the total prevalence of each plasmid type across all 102 clones. Plasmid types are indicated by their incompatibility (Inc) group designations.

Whole-genome sequencing revealed 17 distinct plasmid types. The most prevalent was IncFIB(K), detected in 85.3% of isolates, with particularly high frequencies in ST11 (80.5%), ST15 (100%), ST29 (100%), and ST307 (100%). The second most common type, IncHI1B, accounted for 66.7% of occurrences. Notably, IncFII(pHN7A8) was exclusively associated with ST11—a finding that suggests niche adaptation, as this lineage lacked IncFII(K) (**Figure 2**).

### Capsular serotype and O antigen distribution

**Figure 3:**
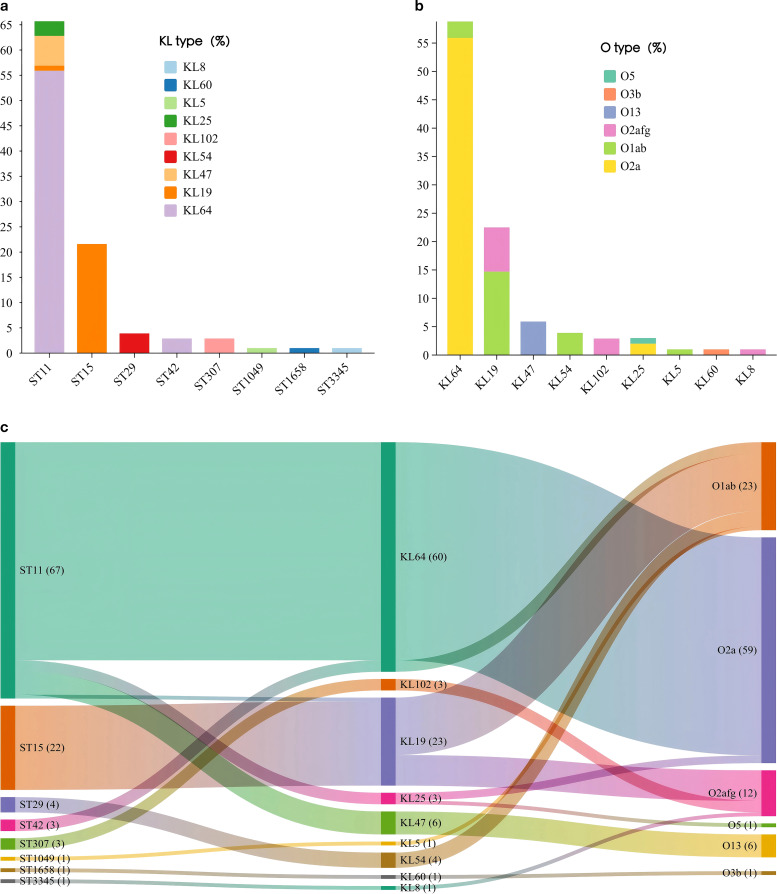
A visual representation of the distribution and associations among different ST, KL, and O types in CRKP isolates in 2022. **(a)** Stacked bar graph illustrates the distribution of various KL types within the strains, categorized by sequence type (ST). Each color in the graph represents one of the nine distinct KL types. **(b)** Stacked bar graph illustrates the distribution of various O types, categorized by KL types. Each color in the graph represents one of the six distinct O types. **(c)** Sankey diagram of the strans with different ST, KL and O types. ST, sequence type; KL, K-locus (capsule type); O, O-locus (lipopolysaccharide type).

Nine distinct capsule-encoding loci (KL) and six O antigen types were predicted based on Kleborate. The most prevalent KL type were KL64 (58.8%, 60/102), followed by KL19 (22.5%, 23/102), with additional types KL47, KL54, KL102, KL25, KL5, KL8 and KL60 being detected (**Supplementary Table 2**). The predominant strain combination was ST11-KL64 (55.9%, 57/102), followed by ST15-KL19 (21.6%, 22/102) and ST11-KL47 (5.9%, 6/102) (**Figure 3a**). O antigen typing revealed O2a (57.8%, 59/102) and O1ab (22.5%, 23/102) as the most common types, with O2afg (11.8%) and O13 (5.9%) also present (**Supplementary Table 3**). A strong KL:O type association was observed, with the KL64:O2a combination (55.9%, 57/102) being exclusively associated with ST11. Of 23 serotype KL19, 22(95.7%) strains belonged to ST15, and 1(4.3%) belonged to ST11. Among KL19 strains, 95.7% (22/23) belonged to ST15, displaying either O1ab (65.2%, 15/23) or O2afg (34.8%, 8/23) antigens. Rare O types O3b and O5 were each found in single isolates of ST1658-KL60 and ST11-KL25, respectively. (**Figures 3b**, **c**; **Supplementary Table 4**).

### Genetic determinants of resistance

Genomic analysis identified *bla*_KPC-2_ as the predominant carbapenemase gene (92.2%, 94/102), with all *bla*_KPC-2_-positive strains demonstrating susceptibility to CZA. Among the remaining strains, five *bla*_NDM_ producers (4.9%) were detected, comprising three *bla*_NDM-5_ isolates belonging to ST307-KL102 and two *bla*_NDM-1_ isolates belonging to ST1658-KL60 and ST3345-KL8, respectively. Additionally, one ST11-KL64 strain was found to co-harbor *bla*_KPC-2_ and *bla*_NDM-1_ (**Supplementary Table 5**).

Notably, we identified two clinically significant *bla*_KPC-2_ variants associated with CZA resistance: (1) *bla*_KPC-33_, containing an A535T nucleotide substitution (GAC→TAC) that results in a D179Y amino acid substitution in the Ω-loop region; and (2) a novel *bla*_KPC-2_ variant with an unreported CTG→ACG mutation at codon 169, leading to an L169T (leucine→threonine) substitution. No *bla*_OXA-48_, *bla*_VIM_, or *bla*_IMP_ genes were detected. Additionally, 85.3% (87/102) of isolates carried mutations in *OmpK35* and/or *OmpK36* porin genes.

**Figure 4:**
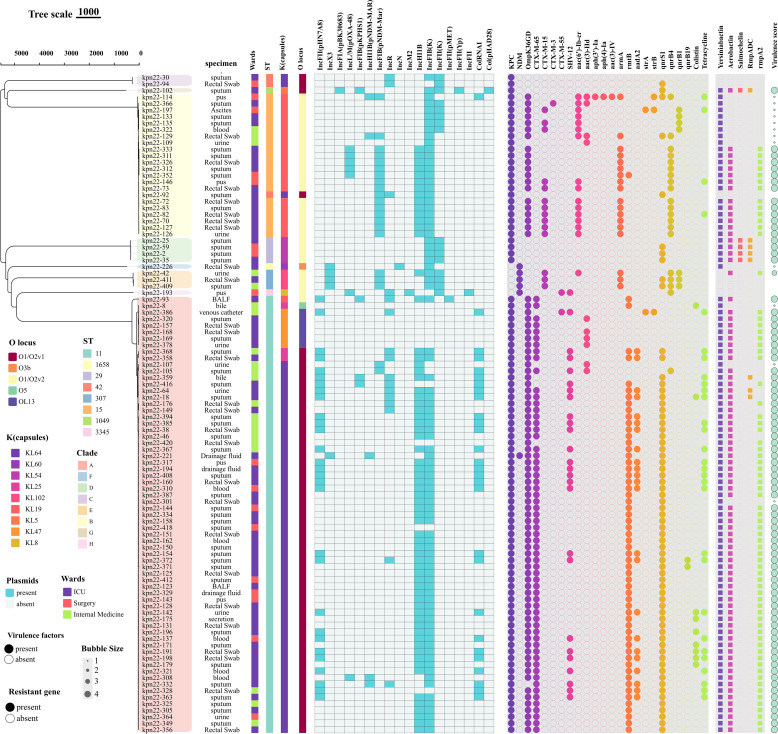
View of phylogenetic analysis and characteristics of 102 CRKP isolates in 2022. The phylogenetic tree was constructed based on core-genome single nucleotide polymorphism SNP analysis. The specimen types were indicated in the column. The wards, sequence type (ST), K-Locus capsule polysaccharide locus (KL), O-Locus lipopolysaccharide (LPS) colours were indicated in the legend. The duck blue square indicate the presence of a plasmid. Resistance genes and virulence factors were indicated by circles and squares, respectively, with different colors representing different types. The green bubble indicated the presence of virulence scores, and the larger the bubble, the higher the score. ST, sequence type; KL, K-locus (capsule polysaccharide type); O, O-locus (lipopolysaccharide type).

The most common cephalosporin (3rd gen.) was *bla*_CTX-M-65_, present in 62 of these CRKP strains, accounting for 60.7%, all belonging to ST-11. Among these strains, 85.4% (53/62) were KL64, 8% (5/62) were KL47, 4.8% (3/62) were KL25 and 1.6% (1/62) was KL19. *bla*_CTX-M-15_ was detected in 13.7% (14/102) of the CRKP strains, among which, eleven belonged to ST15-KL19, and the other three belonged to ST307-KL102. Two *bla*_CTX-M-55_ were detected in each ST11-KL47 and ST3345-KL8, and one *bla*_CTX-M-3_ was in ST15-KL19 (**Figure 4**).

The plasmid-mediated quinolone resistance (PMQR) gene qnr was detected in 87 strains, accounting for 85.3%, with *qnrS1* being most common (70.1%, 61/87) followed by *qnrB4* (17.2%, 15/87). Notably, seven strains carried multiple qnr variants, including two strains with *qnrB4* + *qnrS1*, two with *qnrB19* + *qnrS1*, two with *qnrB1* + *qnrB4* + *qnrS1*, and one with *qnrB1* + *qnrB4* (**Supplementary Table 6**).

### Virulence

In our study, we assessed the distribution of virulence factors associated with the hv*Kp* pathotype, including yersiniabactin, salmochelin, aerobactin, colibactin and hypermucoidy (*RmpADC*/*rmpA2*), and calculated the virulence scores for 102 CRKP isolates.

**Figure 5:**
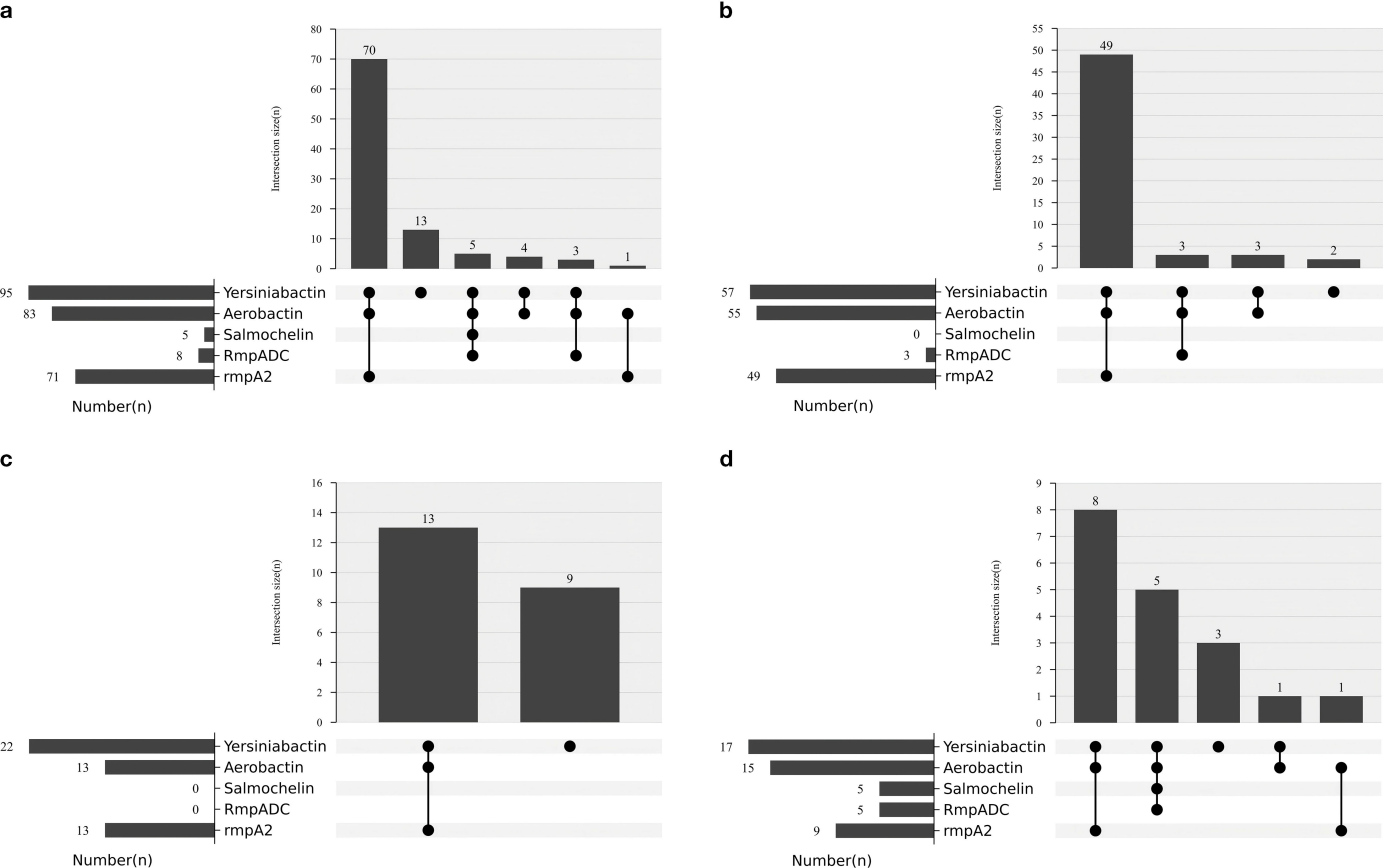
Virulence Distribution. **(a)** An UpSet plot was generated for all the 102 CRKP strains, **(b)** ST11-KL64 strains, **(c)** ST15-KL19 strains, and **(d)** other strains (neither ST11-KL64 nor ST15-KL19) to visualize the presence or absence of the virulence factors, including yersiniabactin (ybt), aerobactin (iuc1), salmochelin (iro1), *rmpADC*, and *rmpA2*. black circles indicate the presence of a given virulence factor. The number above each bar represents the number of strains with that specific virulence configuration. In panel a, strains that lack all the five virulence factors are not shown in the plot.

The yersiniabactin *(ybt*) siderophore gene cluster, which is associated with integrative conjugative elements (ICE*Kp*), was detected in 95 isolates (93.1%, 95/102) (**Figure 5a**). Among these, the ICE*Kp3* lineage (encoding *ybt*9 sequence type) was the most prevalent, identified in 70 isolates (73.7%, 70/95), of which, 81.4%(57/70) were ST11-KL64. And the ICE*Kp12* lineage (encoding *ybt*14 sequence type) was the second common, found in 24 isolates (25.3%,24/95), of which, 91.7%(22/24) were ST15-KL19 (**Supplementary Table 7**). A total of 83 strains (81.4%,83/102) harbored the aerobactin-encoding *iuc1* gene. While the *iro1* gene encoding salmochelin synthesis was rare, found in only five isolates (4.9%), among which, four were ST29-KL54 and one was ST1049-KL5. Additionally, 71 isolates (69.6%) harbored the *rmpA2* gene and 8 isolates (7.8%) carried the *rmpADC* gene, which were associated with hypermucoidity (**Figure 5a**).

The majority of isolates (82, 80.4%) exhibited a high virulence score of 4, according to the Kleborate analysis results, carrying aerobactin and/or salmochelin with yersiniabactin, but without colibactin. A smaller subset of isolates had lower virulence scores: 12 (11.8%) had a score of 1, carrying yersiniabactin only, 1 (1.0%) had a score of 3, which indicated the carriage of the aerobactin and/or salmochelin only, but without yersiniabactin or colibactin, and 7 (6.9%) had a score of 0 (**Figure 4**). Meanwhile, the distribution of virulence factors differs among serotypes of CRKP strains. For example, 91.2% (52/57) of the strains simultaneously carried the aerobactin, yersiniabactin, and *rmpA2* genes in ST11-KL64, while 59.1% (13/22) in ST15-KL19. The virulence profile of ST15-KL19 strains was relatively homogeneous: 59.1% carried aerobactin, yersiniabactin, and *rmpA2*, while the remaining isolates harbored only yersiniabactin. Notably, salmochelin was absent in both ST11-KL64 and ST15-KL19 strains but was detected in ST29-KL54 and ST1049-KL5 strains, which also co-harbored the *rmpADC* gene (**Figure 5**).

### Phylogenetic surveillance of CRKP clinical isolates

To assess the genetic diversity of the CRKP isolates, we performed core-genome SNP analysis on all 102 strains. The phylogenetic tree (**Figure 4**) revealed that the 102 isolates clustered into eight distinct clades (A–H), with approximately 5000 SNPs between different clades, indicating that isolates from different clades are genetically unrelated. Clade A (67/102, 65.6%) and Clade B (23/102, 22.5%) emerged as the predominant lineages. Within the predominant Clade A (ST11, n=67), which includes the 57 ST11-KL64 isolates and 10 ST11 isolates with other KL types, the pairwise SNP distances ranged from 0 to 275 SNPs. This level of intra-clade diversity exceeds the typical threshold for a single clonal outbreak (usually <10–15 SNPs), indicating that even within the dominant clade, these isolates represent multiple independent transmission events rather than a single outbreak.

All Clade A isolates (n=67) belonged to ST11, with 85.1% (57/67) displaying the ST11-KL64-O2a combination. These isolates were primarily recovered from ICU (62.6%, 42/67), followed by Internal Medicine (16 isolates) and Surgery (9 isolates) departments. Notably, 95.5% (64/67) of Clade A strains demonstrated a virulence score of 4, while only three isolates scored 1. All Clade A isolates produced KPC carbapenemase, with one co-producing KPC and NDM. This dual KPC+NDM-producing ST11-KL64 strain uniquely carried an IncX3 plasmid, which was absent in other Clade A isolates.

Clade B predominantly consisted of ST15-KL19 strains (22/23, 95.6%), along with one genetically distinct ST42-KL64-O1ab isolate. The ST42 isolate exhibited a virulence score of 0 and lacked key resistance determinants including *bla*_CTX-M_, *armA*, *OmpK36*GD mutation, and QRDR mutations in *gyrA*/*parC*, distinguishing it from the predominant ST15-KL19 strains in this clade.

Among the remaining five NDM-producing strains, three ST307-KL102-O2afg isolates clustered in Clade E. Notably, one urinary isolate demonstrated a virulence score of 3, while two others from sputum and rectal swab specimens scored 0. The remaining two NDM-producing strains (ST1658-KL60-O3b and ST3345-KL8-O2afg) belonged to Clade F and H, respectively, representing novel ST types not previously reported.

## Discussion

The emergence of CRKP has become a critical public health challenge, particularly in intensive care units where our study found 62.7% of CRKP infections occurred. The predominance of respiratory isolates (51.9%) aligns with CHINET surveillance data (Qin et al., 2024), contrasting with urine-predominant patterns in South America and blood-predominant in the US (Wang et al., 2022), reflecting regional epidemiological variations. More alarmingly, our rectal screening on hospitalized patients in 2022 revealed CRKP colonization in 27.5% (28/102) of patients, with 32.1% (9/28) progressing to bloodstream, urinary or respiratory infections, demonstrating the gastrointestinal tract’s role as a key reservoir for subsequent invasive infections (Bray et al., 2025).

Notably, CRKP resistance declined from 18.5% in 2019 to 13.6% in 2021, then rebounded sharply to 25.0% in 2022. This decline-rebound pattern aligns with findings from another Southwest China hospital (Du et al., 2025), suggesting shifts in infection prevention and control (IPC) measures and antimicrobial pressure across different phases of the COVID-19 pandemic. The initial decline (2020–2021) likely resulted from stringent IPC measures (enhanced hand hygiene, disinfection, isolation, and restricted mobility) that disrupted CRKP transmission (Ficik et al., 2023), and reduced patient turnover reduced patient turnover. The 2022 rebound reflects reversed trends: post-pandemic relaxation of control measures restored hospital capacity and patient mobility. ICUs and respiratory wards faced surges of critically ill COVID-19 patients, straining systems and facilitating CRKP spread (Du et al., 2025; Shi et al., 2025). Concurrently, widespread empirical use of broad-spectrum antibiotics, particularly carbapenems, intensified selective pressure, driving resistant strain proliferation.

The clinical burden of CRKP infections is substantial, with our cohort demonstrating a 32.6% (76/233) in-hospital mortality rate, significantly higher than carbapenem-sensitive infections (Ramos-Castañeda et al., 2018; Li et al., 2023). This excess mortality reflects the convergence of multiple factors: intrinsic multidrug resistance that severely limits therapeutic options (Itani et al., 2025), frequent occurrence in patients with underlying comorbidities (Wang et al., 2025), and the synergistic effects of critical illness markers. Our multivariate analysis identified particularly lethal associations, including multiple organ dysfunction syndrome (aOR 4.362) and sepsis (aOR 3.04), the latter’s pathophysiology involving a dysregulated host response that demands particularly prompt and precise antimicrobial intervention.

Moreover, ICU admission (aOR 4.095) and mechanical ventilation (aOR 5.948) emerged as particularly strong mortality predictors, underscoring the synergistic risk posed by invasive procedures in colonized patients. These findings collectively highlight the urgent need for enhanced prevention strategies targeting: (1) active surveillance of gastrointestinal colonization, (2) antimicrobial stewardship in high-risk units, and (3) reduced utilization of invasive devices in CRKP-colonized patients.

In addition to clinical epidemiology, a thorough characterization of molecular determinants, including virulence factors, plasmid profiles, and capsular serotypes, proves critical for developing effective interventions against CRKP dissemination. Our phylogenomic analyses revealed distinct clone-specific plasmid maintenance patterns in hv-CRKP. Notably, the co-occurrence of plasmids carrying both virulence factors and resistance genes emerged as a key driver of hv-CRKP emergence. Intriguingly, despite the potential for substantial plasmid diversity within individual clones, phylogenomic studies indicate that certain clones stably maintain specific plasmids over extended periods, even during clonal expansion. For example, ST307 has long been associated with the *bla*_CTX-M-15_/IncFIIk plasmid (Wyres et al., 2019), and our findings corroborated this association, as all three ST307 strains belonging to Clade E harbored *bla*_CTX-M-15_ on IncFII(K) plasmids. Similarly, ST11 has been linked to the *bla*_KPC_/IncFII(pHN7A8) plasmid (Li et al., 2022). Such stable plasmid-clone associations likely reflect finely tuned evolutionary adaptations, where specific genetic backgrounds optimize the maintenance and expression of particular resistance-virulence combinations.

The emergence of ceftazidime-avibactam resistance among CRKP isolates represents a significant therapeutic challenge that warrants detailed molecular characterization. Our data demonstrate a concerning rise in resistance rates from 3.1% in 2020 to 9.6% in 2022, driven primarily by two distinct mechanisms: the increasing prevalence of metallo-β-lactamase-producing strains and the mutation of *bla*_KPC-2_. Of particular concern is the *bla*_KPC-33_ variant (D179Y), first reported in China in 2020 during treatment of *bla*_KPC-2_-positive *K. pneumoniae* infections (Shi et al., 2020), which has since been increasingly documented across multiple clinical centers (Lei et al., 2024; Liu et al., 2025).

Molecular characterization also identified a novel KPC-2 variant featuring a CTG→ACG transition at codon 169, generating an L169T (Leucine→Threonine) substitution within the critical Ω-loop domain. To our knowledge, this represents the first documented occurrence of this mutation pattern, which confers resistance to CZA with an elevated MIC of 32 μg/mL. The discovery expands the repertoire of known Ω-loop mutations associated with CZA resistance, including KPC-12 (L169M) and KPC-35 (L169P) with reduced carbapenemase and CZA activity (Hemarajata and Humphries, 2019; Qin et al., 2021). The repeated emergence of mutations at residue suggests L169 serves as a critical mutational hotspot under selective pressure from CZA therapy.

Of particular clinical significance, we identified an ST11-KL64 strain harboring both *bla*_KPC-2_ and *bla*_NDM-1_ genes that exhibited CZA resistance (MIC >32 μg/mL). Whole-genome sequencing revealed the acquisition of an *Inc*X3 plasmid carrying *bla*_NDM-1_, which converted this originally CZA-susceptible strain into a resistant phenotype. This finding highlights the potential for plasmid-mediated co-transfer of metallo-β-lactamases to confer CZA resistance in KPC-producing CRKP strains, representing an emerging resistance mechanism that warrants close surveillance.

In our study, the IncFII(pHN7A8) plasmid was exclusively detected in ST11 strains, suggesting its stable maintenance within this clone through transposon and prophage-mediated integration mechanisms, thereby promoting its dissemination and persistence (Sun et al., 2023). Furthermore, we identified a unique plasmid profile in the ST11-KL64 clone, characterized by a high prevalence (>80%) of IncHI1B and IncFIB(K) plasmids and the absence of IncFII(K), IncFII(Yp), IncFII(pMET), or IncN plasmids. IncFIB(K) emerged as the most common virulence plasmid (Tian et al., 2021), often associated with multidrug-resistant plasmids (Wyres et al., 2020). These findings highlight the distinct plasmid composition of ST11-KL64 and suggest that specific plasmids may enhance the clone’s survival and persistence. Future research should focus on elucidating the specific roles of these plasmids in the pathogenicity and transmission of ST11-KL64, as well as their potential impact on clinical outcomes and antimicrobial resistance patterns.

On the other hands, *K.pneumoniae* strains are serologically characterized by their K-Locus capsular polysaccharide (CPS) and the O-Locus lipopolysaccharide (LPS), which serve as the major virulence determinants that significantly contribute to gastrointestinal colonization (Calderon-Gonzalez et al., 2023). And different serotypes exhibit varying capacities to facilitate immune evasion and bacterial survival during infection (Xu et al., 2024). For example, high-virulence *K. pneumoniae* (hv*KP*) is widely found in serotypes KL1 and KL2, which was known as CR-hvKP when acquired carbapenem-resistance plasmids (Tian et al., 2022; Lei et al., 2024). However, KL1/KL2 CR-hvKP did not cause pandemics. In contrast, CRKP strains involved in acquiring virulence plasmids, resulting in the emergence of hv-CRKP with KL64 and KL47 being the dominant serotypes, both belonging to ST11 (Zhou et al., 2023). Recombinations in the CPS synthesis locus drive genetic diversity, and ST11-KL64 hv-CRKP has now replaced ST11-KL47 as the dominant variant due to its enhanced pathogenicity (Zhou et al., 2020; Wang et al., 2024). In our study, most of the CRKP strains (65.7%) were identified as ST11-KL64, while only 6 isolates were ST11-KL47, further highlighting the dominance and potential spread of the ST11-KL64 variant in the current clinical setting.

The *rmpA/rmpA2* genes, located on the virulence plasmid of hvKp, are key regulators of the mucoid phenotype, promoting capsular polysaccharide synthesis and secretion (Cheng et al., 2010). In our strain collection, *rmpA2* was detected in 69.6% (71/102) of isolates, with the majority of *rmpA2*-positive strains belonging to the ST11 clone, demonstrating the strong association of this virulence determinant with the ST11 lineage. In contrast, the *rmpADC* locus, another important genetic determinant of hypermucoidy, was present in only 7.8% of the CRKP strains. While this prevalence is significantly lower than the 38% reported in Chinese isolates, it remains higher than rates observed in South America (0%) and the United States (<1%) (Wang et al., 2022). These substantial geographical variations suggest distinct regional distributions of virulence determinants, likely reflecting differences in evolutionary pressures or epidemiological patterns that influence the acquisition and maintenance of these virulence genes across different populations.

In addition to capsule production, siderophores including yersiniabactin, enterobactin, salmochelin, and aerobactin represent dominant virulence factors in hvKP, showing stronger association with invasive human infections compared to non-invasive isolates (Zhu et al., 2021). The yersiniabactin (*ybt*), carried by the integrative conjugative element ICE*Kp*, emerged as the most prevalent mobile genetic element(MGEs) linked to virulence (Lam et al., 2018). These elements readily disseminate among *K. pneumoniae* strains and can be spread to other Enterobacteriaceae by horizontal gene transfer (HGT) (Farzand et al., 2021).

Our analysis revealed yersiniabactin in 93.1% (95/102) of isolates, predominantly as the *ybt* 9/ICE*Kp3* variant (73.7%, 70/95), followed by *ybt* 14/ICE*Kp12* (25.3%, 24/95). Notably, all ST11-KL47 strains carried *ybt* 9/ICE*Kp3*, with 96.4% co-harboring aerobactin (*iuc*), which is statistically associated with clinical infections and increased mortality (Wyres et al., 2020). This contrasts sharply with ST11-OXA48-producing strains from Spain and the Netherlands, which predominantly carry *ybt* 10/ICE*Kp4* (Jati et al., 2023), highlighting significant geographical variation in virulence and resistance profiles.

Interestingly, while all ST11-KL64 strains lacked salmochelin (*iroBCDN*), this apparent virulence attenuation was counterbalanced by enhanced antioxidant capacity and macrophage survival (Hong et al., 2024; Jia et al., 2024). This adaptation suggests an evolutionary trade-off where loss of certain virulence genes may confer selective advantages in specific environments, without compromising overall pathogenicity.

In summary, CRKP infections in TCM hospitals pose significant treatment challenges, with high mortality observed in critically ill patients. The predominance of ST11-KL64 and ST15-KL19 clones carrying *bla*_KPC-2_ is particularly concerning, especially with the emergence of KPC variants and acquisition of NDM-encoding plasmids, which confer resistance to CZA. Coupled with their hypervirulent potential, these findings underscore the critical need for enhanced molecular surveillance, strict infection control, and optimized antimicrobial strategies in TCM healthcare settings to combat these multidrug-resistant, virulent strains.

## Data Availability

The datasets presented in this study can be found in online repositories. The names of the repository/repositories and accession number(s) can be found in the article/**Supplementary Material**.
